# Mechanical Characterization of Synthetic Gels for Creation of Surrogate Hands Subjected to Low-Velocity Impacts

**DOI:** 10.3390/gels8090559

**Published:** 2022-09-02

**Authors:** Eduardo M. Sosa, Marta M. Moure

**Affiliations:** 1 Department of Mechanical and Aerospace Engineering, West Virginia University, Morgantown, WV 26506, USA; 2 Aerospace Systems and Transport Research Group, Rey Juan Carlos University, 28942 Fuenlabrada, Madrid, Spain

**Keywords:** human hand, impact test, synthetic gel

## Abstract

The development of human body simulators that can be used as surrogates for testing protective devices and measures requires selecting synthetic materials with mechanical properties closely representative of the human tissues under consideration. For impact tests, gelatinous materials are often used to represent the soft tissues as a whole without distinguishing layers such as skin, fat, or muscles. This research focuses on the mechanical characterization of medical-grade synthetic gels that can be implemented to represent the soft tissues of the hand. Six grades of commercially available gels are selected for quasi-static hardness and firmness tests as well as for controlled low-velocity impact tests, which are not routinely conducted by gel manufacturers and require additional considerations such as energy level and specimen sizes relevant to the specific application. Specimens subject to impacts represent the hand thicknesses at the fingers, knuckles, and mid-metacarpal regions. Two impact test configurations are considered: one with the gel specimens including a solid insert representing a bone and one without this insert. The impact behavior of the candidate gels is evaluated by the coefficient of restitution, the energy loss percentage, and the peak reaction force at the time of impact. The resulting values are compared with similar indicators reported for experiments with cadaveric hands. Relatively softer gels, characterized by Shore OOO hardness in the range of 32.6 ± 0.9 to 34.4 ± 2.0, closely matched the impact behavior of cadaveric specimens. These results show that softer gels would be the most suitable gels to represent soft tissues in the creation of surrogate hands that can be used for extensive impact testing, thus, minimizing the need for cadaveric specimens.

## 1. Introduction

Hand injuries are a major problem in several industries. Impacts produced over the hands are common in production settings, in which hand injuries constitute a non-negligible portion of total injuries, particularly in extractive or manually intensive industries. These injuries are often correlated to the use of machinery, material handling, and falling objects, which can produce wounds with varying degrees of severity with the consequent functional limitations. The resulting hand disabilities often require adjustments of work responsibilities, retraining, or even applying for permanent disability benefits [1,2,3,4].

In the past years, experimental studies with cadaveric hands have been completed to measure the forces resulting from low-velocity impacts (typically ≤ 5 m/s) on different regions of the hand [5,6,7]. While those tests provide valuable information regarding the order of magnitude of the forces resulting from the impact, they can be limited by the number and variability of testing specimens prompting the development of surrogate hands that can closely mimic the impact behavior of real hands.

Surrogate specimens representing different human body parts are used in various fields for a variety of purposes. For example, medical residency programs utilize realistic simulators for training purposes. Some examples of this application include a hand simulator for the training of percutaneous pinning [8], a patient-specific model to mimic a clubfoot [9], a model to create a shoulder simulator [10], and a simulator of vasculature components included as part of an extracorporeal cardiopulmonary resuscitation training manikin [11]. On the other hand, surrogate specimens of human parts are commonly used to evaluate protective measures for different situations. For example, automobile crash safety tests use standardized full or partial body dummies [12], and the performance of helmets has been evaluated with specialized head forms [13,14].

The soft tissues of those body dummies are often represented by gel-like materials such as synthetic gels. Synthetic gels are three-dimensional (3D) networks composed of elastic polymer molecules and fluid. Any fluid, including water (hydrogels), oil, and air (aerogel), could be used to create a gel. The combination of molecules and fluid filling the interstitial spaces confers the capability of undergoing substantial deformations to the 3D network [15]. In the formation of the gel, the solid polymer chains swell in the liquid matrix but do not dissolve in it. This effect enables the network to exhibit viscoelastic properties attractive for biomedical and other similar applications [15]. Synthetic gels can also be produced at a mass scale and have superior mechanical and chemical stabilities over natural water-based gels such as hydrogels. Compared to natural gels, synthetic gels have higher reproducibility in terms of physicochemical and mechanical properties [15].

Some typical synthetic gels implemented for tissue engineering include polyethylene glycol (PEG) [15] and polyvinyl alcohol (PVA) [16]. Other synthetic gels include poly-hydroxyethyl methacrylate (PHEMA), used in pharmaceutical applications, and Polyurethanes (PU), which are used in biomedical applications due to their ability to act as a thermoplastic elastomer. Hydrogels have been studied extensively for drug release and tissue engineering [16,17,18,19,20,21] and for monitoring human health [22].

**Figure 1:**
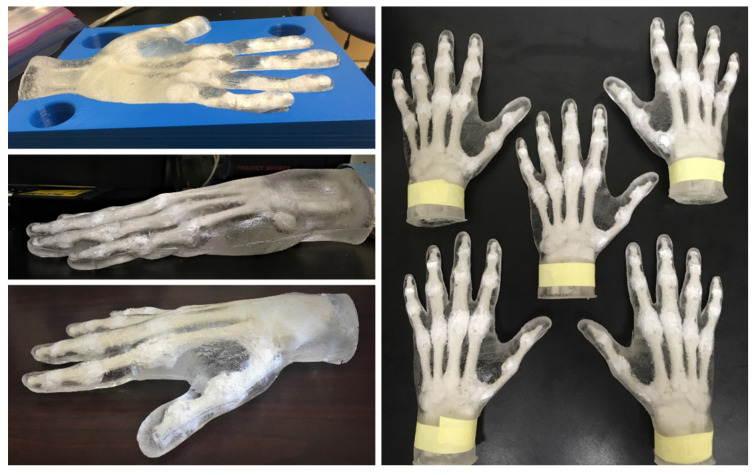
Lateral and top views of surrogate hand specimens described in [27].

On the other hand, oil-based synthetic gels often combine mineral or natural oils with polymers such as styrene–ethylene–propylene or styrene–ethylene–butylene–styrene, among other polymers, to achieve different gel characteristics suitable for mimicking different types of soft tissues [23,24]. These gels have been used to create ultrasound phantoms, typically for calibration of ultrasound equipment and medical training [25] and cardiovascular flow phantoms [26]. More recently, the authors assessed the impact behavior of protected and unprotected surrogate hands [27]. In that study, surrogate hands were built as a combination of synthetic gels representing the soft tissues as a whole and surrounding a 3D-printed bone structure and used to assess the impact protection of typical metacarpal gloves. The surrogate hand specimens (shown in Figure 1) were subjected to controlled impact tests, and their calibration and validation were based on impact response data reported previously for cadaveric hand specimens [7].

A key aspect of the realism of the simulators comprised of gel materials is having a stiffness similar to the actual soft tissues they represent. However, gel materials are challenging to characterize using conventional testing methods due to the low internal forces that these materials can withstand. A recent study found that different testing modalities do not necessarily produce the same mechanical measurements within a material [28]. This characteristic is more evident for high to very-high loading (or strain) rates in which the mechanical behavior is a function of the strain rate. The material exhibits a higher stress response when the strain rate increases [29,30].

Different methods have been used in the past to evaluate the static response of gel materials, including indentation [28,31], tension [32], and compression [21,28,32] tests. The dynamic response under different loading rates was evaluated using eccentric transient rotational impacts [31], linear shock machines [33], as well as using Split Hopkinson Pressure bars (SHPB) for very high strain rates [29].

This study presents a systematic evaluation of the mechanical response of synthetic gels to a series of quasi-static tests and controlled impact tests to determine their firmness, hardness, and reaction against low-velocity (~2 m/s) impacts. The experimental work performed on cadaveric specimens subjected to controlled impact tests was reported in [7] and served as the basis for the development and testing of surrogate hands reported in [27]. The synthetic gels evaluated in this study correspond to the material candidates considered for creating surrogate hands that can be used for systematic impact testing reported in [27]. The hardness, firmness, and impact characteristics of the gel materials were not reported previously, and they are the focus of the current research.

A description of the main characteristics of the gels and the main features of the tests are presented in Section 2. The test results and observations are presented in Section 3, and finally, Section 4 presents the main conclusions of this research.

## 2. Materials and Methods

### 2.1. Materials: Synthetic Gels

Oil-based, medical-grade, 100% synthetic, and clear gels produced by Humimic Medical (Greenville, SC, USA) [34] were selected for testing. This type of gel is commonly used for making tissue mockups typically used in medical training. The gels are comprised of a combination of oils and gellants. The proportion of each component ranges from 75% to 95% for the oil, and from 5% to 25% for the gellant, depending on the desired flexibility needed to simulate a specific soft tissue [34]. The gels come in 1-pound (0.454 g) bags and are ready to be used. The gels do not require special preparation other than heating in a conventional oven until they reach the melting point (92 °C to 120 °C) needed to pour them into molds. After the melted gel cools down and solidifies, it remains stable at room temperature and is odorless. This study considered six gel grades (#0 to #5) to identify the grade that most closely resembles the impact behavior of unprotected hands subjected to impact tests reported in the literature [6,7]. Table 1 summarizes their densities and potential uses for mimicking different soft tissues, as reported by the manufacturer [34].

### 2.2. Quasi-Static Material Tests

**Figure 2:**
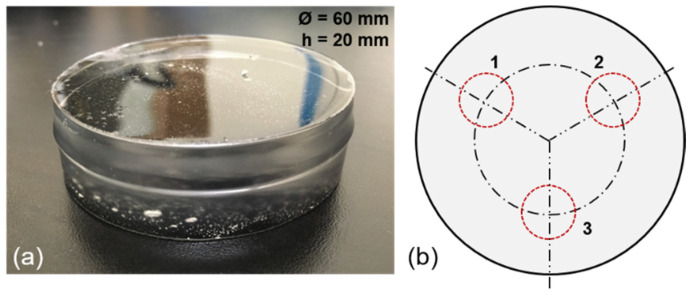
(**a**) Cylindrical test specimen used for firmness and hardness tests; (**b**) Probing locations and probing sequence.

Two sets of quasi-static tests were conducted to initially characterize the mechanical behavior of candidate gels, including:

Hardness tests: The hardness of the candidate gels was determined by following the ASTM D2240:2021 standard [35]. Sets of three cylindrical-shaped specimens for each grade of gel were manufactured for this test. Each specimen was 60 mm in diameter and 20 mm thick, as shown in Figure 2a. After pouring the gel into the cylindrical mold, the specimens were cooled down and maintained at a constant room temperature (23 °C) for 24 h, after which the hardness was measured. The hardness was measured with a Rex durometer (Rex Gauge Co., Buffalo Grove, IL, USA) calibrated for the Type OOO scale coupled with a fixed 400-g mass aligned with the indenter. The Shore OOO scale is typically used to measure the hardness of rubber materials and gels that are very soft. The scale ranges from 0 to 100, which corresponds to the spring forces of the durometer ranging from 0.203 to 1.11 N. As a reference, more rigid rubbers are measured using Shore A or D scales in which the spring forces of the durometer range from 0.55 N to 8.05 N and from 4.45 N to 44.45 N, respectively [35]. Average values and standard deviation (SD) were calculated using data obtained from three readings for each specimen following a triangular pattern illustrated in Figure 2b.

Firmness tests: Like in the hardness tests, the firmness was tested using cylindrical specimens with the same dimensions used for the hardness tests. Gel specimens were manufactured from batches of new gel poured into the molds, then allowed to cool down and reach a constant room temperature (23 °C) for 24 h. A 12.7 mm in diameter stainless-steel probe with a flat contact surface was selected for the tests. This probe was mounted in a Shimadzu EZ-LX universal testing machine equipped with a 500 N load cell and set to produce a quasi-static uniaxial compression. After demolding, the specimens were placed on top of a flat surface fixed to the lower support of the testing machine. The cylindrical probe was then placed near the top surface of the specimen without contacting it and maintaining a separation of 1 mm. The probe was then set to travel vertically at a 25 mm/min (0.42 mm/s) rate until it was inserted 4 mm into the specimen. The probe was returned to its initial position when this limit was reached. The firmness value was determined by the peak force necessary to produce the 4 mm penetration. This procedure was repeated three times for each specimen so the probe could be inserted at three locations on the surface following a triangular pattern, as illustrated in Figure 2b. Values obtained from these tests were compared to values reported by the manufacturer [34].

### 2.3. Impact Tests

**Figure 3:**
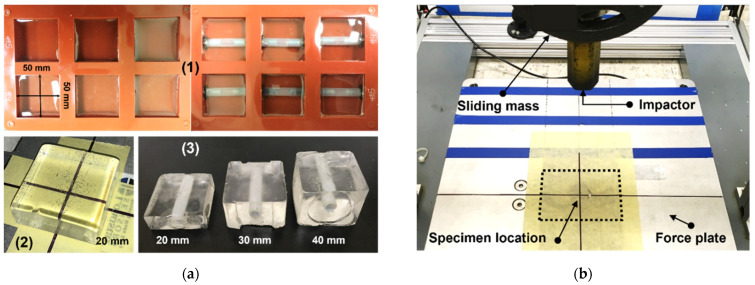
(**a**) 1—Molds for prismatic specimens without and with tubular inserts; 2—20 mm plain gel test specimen; 3—Specimens with tubular inserts; (**b**) Impact test setup.

Controlled impact tests were conducted to determine the gel response to a falling mass. Key features of the impact tests included:

Specimens: Sets of three prismatic specimens (50 mm by 50 mm) for each candidate gel were manufactured for the impact tests. Each set included three thicknesses representative of the hand depths at the fingers (20 mm), knuckles (30 mm), and mid-metacarpal region (40 mm). The specimens were prepared from batches of gel material poured into the molds (Figure 3a-1), then allowed to cool down and reach a constant lab room temperature (23 °C) for 24 h before testing. Two specimen configurations were tested: one with plain prismatic gel specimens and a second one with prismatic gel specimens, including a tubular insert, as shown in Figure 3a-2 and Figure 3a-3, respectively. This tubular insert had a 10 mm outer diameter and an 8 mm inner diameter, and it was intended to simulate the presence of a bone. The tubular inserts were fixed to the molds by two thin metallic washers placed at each end of the tubular insert that served as anchors during the gel pouring. These washers had a variable diameter necessary to maintain the tubular insert in the middle of the thickness of the specimens during and after pouring the gels. Once the gel solidified, the washers were carefully removed, and the tubular insert remained in place at the center of the specimen thickness, as shown in Figure 3a-3. This tubular insert is made of nylon material, which is much stiffer than the gels considered in this study and similar in terms of mechanical properties to the nylon material implemented in [27] to reproduce human finger bones. This material had a hardness value of R110 (Rockwell scale). It can withstand up to 150 °C, which is above the melting gel temperature (max 120 °C), so the insert does not get distorted or damaged when pouring the melted gel.

Impact testing machine: A custom-made guillotine-type impact testing machine was used for controlled impact tests. The device included a metallic mass (5 kg) set to slide vertically. This mass was coupled to a metallic impactor with a hexagonal cross-section, a flat striking surface, and an outer diameter of 32 mm. The mass of the impactor, its shape, and its size are the same as implemented by [7,27] in their study of impacts on cadaveric hands. The instrumentation included: (a) A 5 kN-capacity force plate (Loadstar Sensors, Fremont, CA, USA) placed underneath the striking impactor to capture the vertical reaction force originated by the impacts; (b) A string potentiometer attached to the sliding mass for recording the displacement of the impactor (ISP-125, Loadstar Sensors, Fremont, CA, USA). The force and position data were recorded at a frequency of 1 kHz and automatically synchronized using the SensorVUE data acquisition software (Loadstar Sensors, Fremont, CA, USA). Figure 3b shows an overview of the testing setup.

Testing procedure: Individual gel specimens were positioned on top of the force plate with their top surface center point aligned with the vertical axis of the impactor. The testing frame held the mass until a signal was triggered for releasing the sliding mass. The drop height (*h*_0_) was set at 200 mm, measured from the surface of the force plate to the striking edge of the impactor. Once the impactor was released and impacted the specimen, the vertical reaction force history was measured directly underneath the specimen’s impact zone by the force plate. The peak reaction force (PRF) was extracted for analysis.

**Figure 4:**
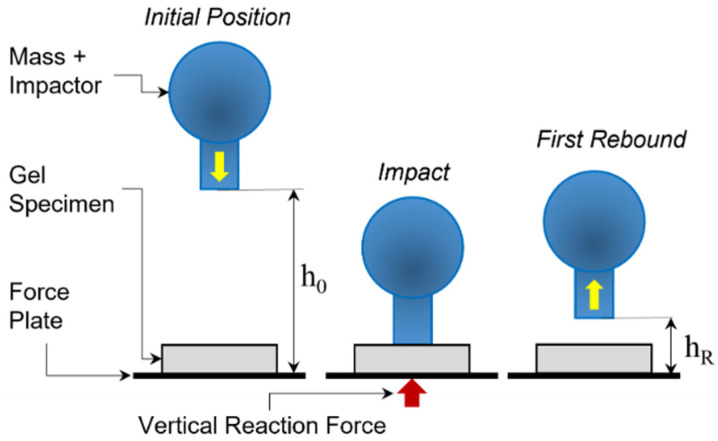
Schematics of impact model.

The impact behavior of the candidate gels was characterized by the Coefficient of Restitution (COR) and the percentage of Energy Loss (EL) of the impactor. The position of the impactor tracked by the displacement sensor was used as input to calculate COR and EL for each gel grade and specimen configuration. The COR was calculated as a relationship between the height of the impactor at the first bounce (*h_R_*) and the starting drop height (*h*_0_), as defined in Equation (1). The EL due to the collision of the impactor and the gel is a function of COR, and it was calculated according to Equation (2) [36,37,38]. Figure 4 provides a schematic representation of the impact components and elevations used for the calculations. The COR ranges from 0, for perfectly plastic impacts, to 1 for perfectly elastic impacts. Considering the relatively small drop height and that all the tests were carried out in a controlled lab environment, the effects of air drag were neglected.

(1) $$\mathrm{COR}=\sqrt{\frac{h_{R}}{h_{0}}}$$

EL = 100 × (1 − COR^2^) (2)

where: *h*_0_ is the initial drop height; *h_R_* is the rebound height; COR is the coefficient of restitution; EL is the energy loss. In this study, impactor displacement history was analyzed to determine the rebound height for the calculation of COR and EL values for all six gel grades, three specimen thicknesses (20, 30, and 40 mm), and two specimen conditions (plain gel and specimens including a tubular insert). Average values and standard deviations were calculated for each combination, and the results are presented in Section 3.

## 3. Results and Discussion

### 3.1. Hardness and Firmness Tests

Hardness tests are often used to determine the properties of a material displaying substantial resistance to deformation. The ASTM D2240 standard [35] is commonly adopted for determining the indentation hardness of materials classified as thermoplastic elastomers, elastomeric materials, and gel-like materials, among others [35]. The Type OOO scale was selected for determining the hardness of the gels considered in this study. The standard recognizes hardness values below 20 and above 90 are unreliable and recommends using the next lower or higher scale type. Preliminary trials with the Type OOO scale indicated that hardness values for the candidate gels were within those limits. Table 2 summarizes the average hardness values obtained from the tests performed on the candidate gels. Values ranged from 51.5 for gel #0 to 24.9 for gel #5. Gel #3 and #4 have similar average values and would have nearly the same hardness if the SD is considered. The hardness ratios calculated with respect to the hardness of gel #0 ranged from 1.00 to 0.48, following an approximately linearly decreasing progression. The hardness ratios show that gel #5 is about half as hard as gel #0 and that gels #3 and #4 are more similar to each other than gel #2 is to gel #1.

On the other side, for soft materials like gels or organic materials, firmness tests are often conducted to measure the resistance to deformation or flow. A common practice is to use penetrometry in which a solid object is pushed into a semi-solid/semi-liquid material sample to measure the force at a single penetration rate [28,31,39,40,41,42,43]. Table 3 summarizes the results of the firmness tests undertaken in the present study compared to the values reported by the manufacturer [34]. The results show that the firmness decreases as the grade of the gel increases, in which gel #0 required the highest force (8.95 N) and gel #5 required the smallest force (1.29 N) to produce a penetration of 4 mm. The firmness ratio calculated with respect to gel #0 ranged from 1.00 to 0.14, following an approximately linearly decreasing progression. As a reference point, a recent study found that the normal force to produce a low-frequency (0.2 Hz) penetration of 2.5 to 3.0 mm is in the range of 0.8 to 1.2 N for a wrist in a neutral position (0°) and for a wrist at 45°, respectively [44]. Measurements reported in [44] were obtained from in vivo dynamic indentation to quantify the compressive characteristics of palmar soft tissues and showed a significant variability with coefficients of variation (COV) in the range of 39% to 110%, which are indicative of the variability in the mechanical behavior of in vivo tissues. This range of indentation forces is in the same order of magnitude as the force measured for gels #3 and #4 (Table 3). However, it must be noted that the force range measured in [33] was obtained with a 3 mm diameter probe, while in this study, the forces were obtained using a 12.7 mm probe. 

Further measurements were carried out with additional gel specimens to determine the effect of the probe diameter on the penetration force. A set of new firmness tests was carried out using a 3 mm-diameter probe with a flat end and a set of new gel specimens. Results summarized in Table 3 indicate that the reduction in probe diameter from 12.7 mm to 3.0 mm reduced the penetration force about five times for all the gels. This reduction is attributed to the needle-like behavior of the 3.0 mm probe, in which significantly less force is necessary to produce a 3 to 4 mm indentation. Considering that the current study is focused on evaluating the behavior of the gels under an impact of an abject with diameters in the range of 20 to 35 mm, the results predicted by the 12.7 mm probe were considered more adequate for better differentiation of the gel stiffness, particularly the ones in the softer range (#3, #4, and #5).

It is also worth noting that cadaveric tissues are often used in biomechanics to determine different mechanical properties that can be used for simulators. Several previous studies found minor variations in the postmortem mechanical properties of bones, ligaments, tendons, and skin [45,46,47,48,49,50] with respect to their live properties. However, for postmortem muscles, the mechanical properties of postmortem specimens displayed significant variations [51,52]. While the results reported in [44] provide valuable information on the order of magnitude of the forces, further similar in vivo studies would be necessary to obtain indentation values covering not only the palmar side of the hand but also at other positions of the hand. Moreover, similar to the observations made in previous studies with different gels [28,29,30], in vivo tests are also highly dependent on the loading rate. Results reported in [44] indicated that the difference between high rate (20 Hz) and low rate (0.2 Hz) loading can be up to three times higher. These results emphasize the importance of measuring tissue (and gels) properties at loading rates close to the application under consideration to reflect the potential for increased loading rate sensitivity present in ordinarily hydrated and live tissues compared with cadaveric tissues [44].

### 3.2. Impact Tests

**Figure 5:**
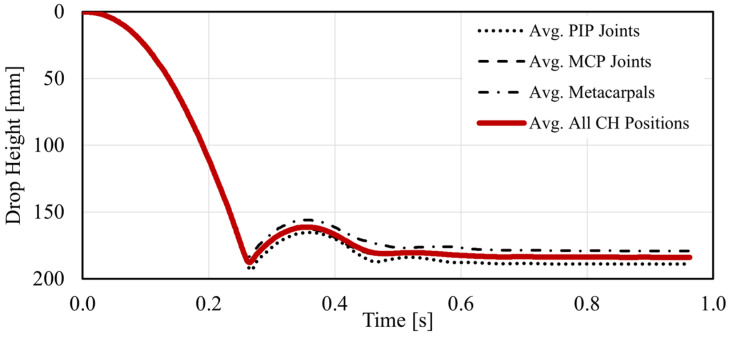
Impactor trajectory obtained from controlled impact tests with cadaveric hands (CH). PIP corresponds to proximal interphalangeal joints; MCP corresponds to metacarpophalangeal joints. Full details can be found in [7].

A total of 108 controlled impact tests were performed in this study. Half of those were carried out with plain prismatic specimens manufactured with six gel grades (gel #0 to gel #5) and three thicknesses (20, 30, and 40 mm). The other half included a 10 mm diameter tubular insert to resemble the presence of a bone. For each test, the falling and bouncing trajectory of the impactor was recorded, and the data was used to determine the COR and EL for each gel type and specimen thickness. The average of those trajectories was compared to the results reported by Sosa & Alessa (2021) [7]. In that study, controlled impact tests were carried out using a set of cadaveric specimens aged 38 to 66 years (avg. 54 years). The average trajectories for impacts on the proximal interphalangeal (PIP) joints, the metacarpophalangeal (MCP) joints, and the mid-length of the metacarpal bones are illustrated in Figure 5. These curves display a single rebound and then stabilize around the hand thickness (typically smaller at the PIP joints than in the metacarpal region) at the impact position of the cadaveric hand (CH) specimens. The average for three impact positions was calculated and used to compare the tests performed with the gel specimens.

**Figure 6:**
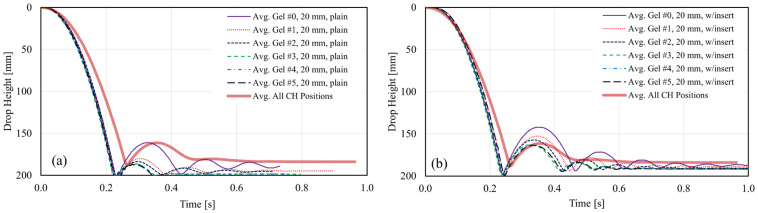
Comparison of average impactor trajectories for 20 mm thick gel specimens: (**a**) plain specimens; (**b**) with tubular insert.

**Figure 7:**
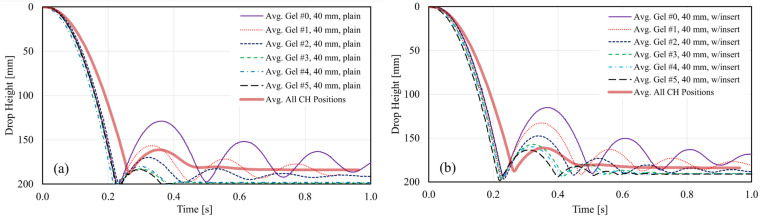
Comparison of average impactor trajectories for 40 mm thick gel specimens: (**a**) plain specimens; (**b**) with tubular insert.

Figure 6 and Figure 7 illustrate average impact trajectories for 20 mm and 40 mm thick specimens, respectively, with and without the tubular insert, and compared to the average curve shown in Figure 5. The first observation is the difference in slopes corresponding to average CH specimens and gel specimens during the initial drop. This difference is attributed to a slight difference in the weight of the sliding mass attached to the impactor and the friction of the sliding components of the testing machine. This difference in slopes translates into a slightly higher impact velocity and kinetic energy (1.82 m/s and 8.47 J, respectively) for the gel specimens than the average values obtained for the CH specimens (1.74 m/s and 7.73 J, respectively [7]). A similar difference was observed for the 30 mm gel specimens as well. Despite this difference, the comparison between the values obtained with CH and gel specimens is still possible to identify a gel type that could mimic the behavior of the soft tissues of the hand.

**Figure 8:**
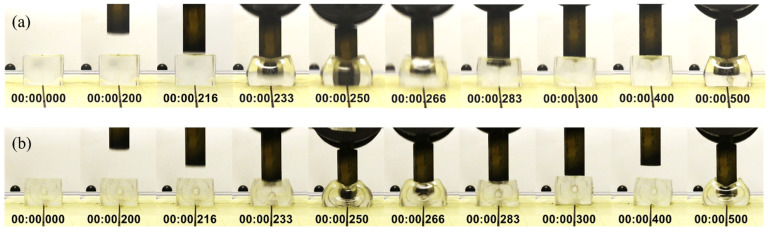
(**a**) Impact sequence for gel #3, 40 mm thick; (**a**) Plain specimen; (**b**) Specimen with tubular insert. Time in min:sec.milliseconds.

**Figure 9:**
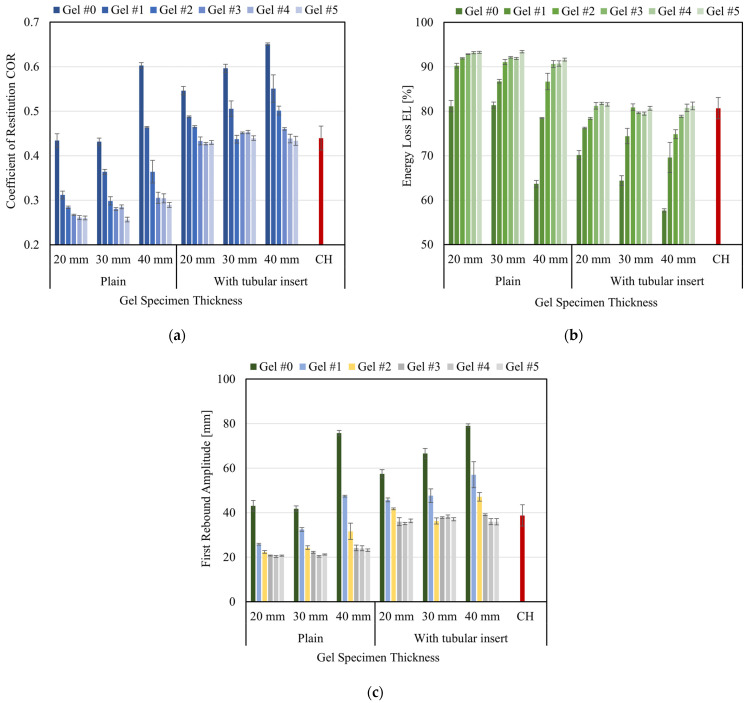
(**a**) Average COR values; (**b**) Average EL values; (**c**) Average elevation of the first rebound. CH values were obtained from [7]. The top error bars correspond to the standard deviation.

The second observation is the number of bounces that followed the initial impact. Stiffer gels such as gels #0 to #2 displayed at least three bounces after the initial impact for plain gel specimens. Softer gels, such as gels #3 to #5, had just one bounce after the initial impact. All plain specimens exhibited a nearly hyperelastic behavior during the first impact and seemed fully penetrated by the impactor, as illustrated in Figure 6a and Figure 7a. Hyperelastic behavior is typical of rubber-like materials subjected to mechanical loading, usually tension or compression. Under these loading conditions, these materials typically exhibit large deformations before breaking and display very large strains, with a strong non-linear stress-strain relationship [53]. For the 40 mm thick specimen, this behavior is also seen in the sequence of images in Figure 8 at t = ~0.25 s. The thickness of the gel specimens appeared to influence the amplitude and number of the bounces that followed the initial impact, especially for stiffer gels (#0 to #2). For these gels and both specimen configurations, twice the thickness (20 mm to 40 mm) resulted in bounces of about ~1.5 to nearly ~2.0 times higher than those seen in 20 and 30 mm thick specimens, as shown in the amplitudes of the first rebound extracted from the trajectories plotted in Figure 6 and Figure 7 and summarized in Figure 9c.

Including the nylon tubular inserts mimicking the presence of a bone had two main effects. The first was to produce an impact behavior that better approximated the behavior seen in the experiments with the CH specimens, as shown in the impactor trajectories of Figure 6b and Figure 7b. The hardness of the tubular insert did not allow full penetration of the impactor, and the position of the impactor stabilized about 10 to 15 mm above the contact surface between the gel specimen and the force plate. This distance roughly corresponds to the outer diameter of the insert. This effect is also illustrated in the sequence of images in Figure 8. The second effect of the tubular insert was a better distinction of the bouncing characteristics of each gel, particularly for the thicker specimen (40 mm), for which softer gels (#3 to #5) seem to reproduce better the behavior seen for CH specimens, as illustrated on Figure 7b.

The impact behavior described previously was quantified by the COR defined in Equation (1) and EL defined in Equation (2). Figure 9a,b illustrate COR and EL results, respectively, as a function of the specimen thickness and testing condition.

From Figure 9a, it is seen that for all the testing thicknesses (20, 30, 40 mm) and specimen configurations (plain and with insert), the overall tendency is that COR values decreased as the hardness of the gel decreased (or the grade of the gel increased). That is, less rigid gel material behaved less elastically and deformed more during the impact, resulting in more kinetic energy converted into deformation energy and, therefore, in smaller rebounds, translating into smaller COR values. The results show that for plain gel specimens, the average COR values were 0.303, 0.319, and 0.388 for 20 mm, 30 mm, and 40 mm thick specimens, respectively. Stiffer gels (#0 to #2) resulted in distinctively higher values than softer gels (#3 to #5), which displayed similar values for their respective thicknesses. On the other side, adding the tubular nylon insert resulted in an overall increase in COR values. The results show that the average COR values for specimens with the tubular insert were 0.465, 0.481, and 0.506 for 20 mm, 30 mm, and 40 mm thick specimens, respectively. These average values are in the range of the average COR value (0.440, SD 0.027) obtained from the experiments with CH specimens. However, only softer gels (#3 to #5) were within the limits of the standard deviation of the CH results. As seen in the plain gel specimens, softer gels resulted in similar COR values, regardless of the specimen thickness. The increase in thickness increased COR values for both types of specimens, plain and with insert. This result is more evident in stiffer gels (#0 to #2), as shown in Figure 9a.

Results summarized in Figure 9b show that for all the testing thicknesses (20, 30, 40 mm) and specimens’ configurations (plain and with insert), the overall tendency is that EL values increased as the hardness of the gel decreased (or the grade of the gel increased). For 20 and 30 mm plain specimens, EL ranged from 81.1% to 93.2 for gel #0 and gel #5, respectively, while for 40 mm specimens, values ranged from 63.7% to 91.6% for the same gels. Plain specimens made of softer gels (3# to #5) displayed a plateau in EL values around 92%, regardless of the specimen thickness.

The inclusion of the tubular insert resulted in an overall reduction of the energy loss, as part of the impact energy is transferred to the tubular insert, whose higher relative stiffness produces an increase in the rebound height of the impactor. This effect translated into higher COR values, as seen in Figure 9a, and a reduction of EL values in Figure 9b. For specimens with the tubular insert, EL values ranged from 70.1% to 81.5% (for 20 mm specimens), 64.4% to 80.7% (for 30 mm specimens), and 57.7% to 81.2% (for 40 mm specimens). EL values for softer gels (#3 to #5) reached a plateau of around 80% for all three specimen thicknesses. Conversely, the average EL value for CH specimens calculated from the curve shown in Figure 5 is 80.7% (SD 2.4). Here again, EL values corresponding to softer gels (#3 to #5) resulted within the range of the CH standard deviation, as illustrated in Figure 9b.

**Figure 10:**
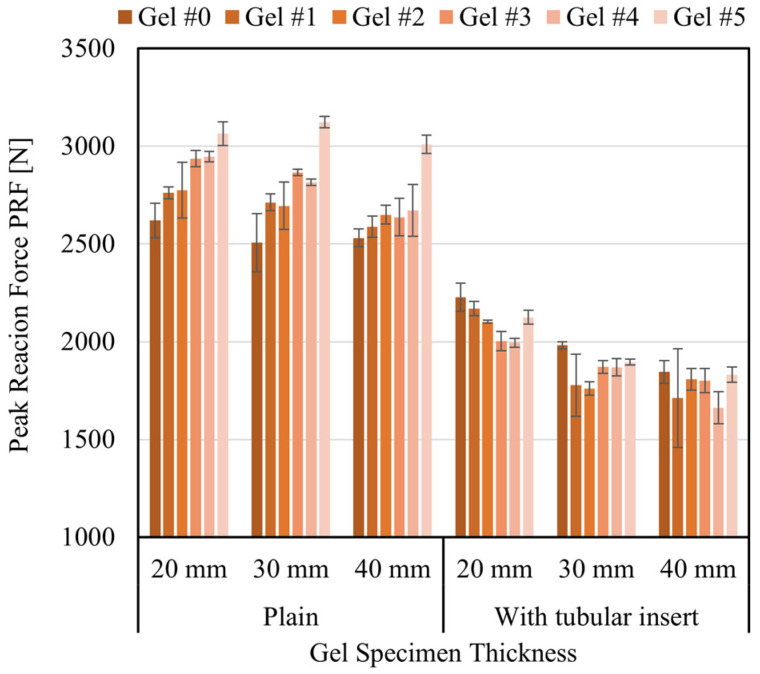
Compilation of average PRF values. The top error bars correspond to the standard deviation.

The third indicator utilized for measuring the impact behavior of the gels was the vertical PRF measured by the force plate. Figure 10 summarizes the average values obtained from all the specimen thicknesses’ (20, 30, and 40 mm) and configurations (plain and with tubular insert). The results shown in Figure 10 indicate that for plain specimens, the overall tendency was to increase PRF values as the gel hardness decreased (or gel grade increased), regardless of the specimen thickness. The softest gel (#5) resulted in the highest PRF values, and all the plain specimens were practically disintegrated after the impacts, which correlates with the low COR values and the high EL values seen in Figure 9a and Figure 9b, respectively. The PRF values ranged from about 2500 N for gel #0 to about 3100 N for gel #5.

The inclusion of the tubular insert produced a reduction in the magnitude of the PRF, as illustrated in Figure 10. This reduction was expected as the stiffness of the insert contributes to the dampening of the impact force. Contrary to plain specimens, the PRF appeared to decrease as the hardness of the gel decreased (or grade increased). This behavior is more evident in the 20 mm thick specimen than in the other two thicknesses, which displayed more variability and similar force magnitude around 1800 N. Also, the PRF values tended to decrease as the thickness of the specimens increased. This behavior is more evident in specimens of stiffer gels (#0 to #2) than softer gels (#3 to #5). Here again, specimens made with gel #5 displayed considerable fragility and practically disintegrated after the impacts. The variability in the PRF values seen in the 30 and 40 mm thick specimens is attributed to the possible presence of imperfections in the vertical position of the tubular insert. It is speculated that a slight inclination of the insert with respect to the specimen’s vertical axis, or eccentricity with respect to the specimen thickness, may have affected the overall response of the specimen during the impact. Thicker specimens (30 or 40 mm) seemed more susceptible to these possible imperfections than thinner specimens (20 mm), as seen in Figure 10. Another important observation regarding the PRF values is the overall reduction of the force magnitude seen in specimens with the tubular insert compared to plain gel specimens. The inclusion of the nylon tubular insert resulted in a decrease of about 31% in the magnitude of the PRF.

It is worth highlighting that the magnitude of the PRF reduction can be tuned by selecting an insert with different material characteristics. That is, higher or lower elasticity, or for the same material but with other geometric properties, such as thicker or thinner walls or different outer or inner diameters. As a reference, recent controlled impact tests conducted with cadaveric hands found that for low-energy impacts (avg. 7.7 J), the force to produce fractures ranged from 1639 N to 2481 on the metacarpal region and PIP joints of the hand, respectively [7]. However, these values can change as a function of age or bone health condition [54], so it becomes evident the need to create reliable surrogate hands that can be used systematically for testing protective measures or fracture patterns resulting from impacts, and thus, minimize the use of cadaveric specimens. Reliable surrogate models can be achieved by the appropriate combination of gel stiffness and solid materials, which, working jointly, can closely mimic the mechanical behavior of the hand’s soft and hard tissues.

Considering the overall results presented for three indicators (COR, EL, and PRF) selected for characterizing the impact response of the different gel grades, it becomes evident that stiffer gels (#0 to #2) are not appropriate for reproducing the impact behavior of soft tissues. These gels displayed a relatively low level of energy loss, which translated into a relatively high level of bouncing after the initial impact. On the contrary, softer gels (#3 to #5) displayed a relatively high level of energy loss and considerably less bouncing than stiffer gels. Among the softer gels, gel #5 demonstrated to be very delicate to handle and fragile against an impact. In contrast, gels #3 and #4 presented a behavior comparable to that seen in previous experiments with actual hand specimens, making them the most likely gel grades to be selected to create surrogate hands for systematic impact testing.

## 4. Conclusions

This study presented the results of a series of quasi-static and dynamic tests carried out to characterize the stiffness of synthetic gels. These gels are used to mimic soft tissues in different medical training applications. They are evaluated to determine their suitability to represent the soft tissues of surrogate hands that can be used for systematic mechanical testing. Specimens of six grades of gels were subjected to firmness and hardness tests to acquire an initial measure of the force levels necessary to deform them. These initial measurements were followed by a series of controlled impact tests designed to measure the ability to dissipate kinetic energy resulting from an impact. The restitution coefficient, energy loss, and peak reaction forces were used as indicators of the performance of gel specimens with and without a tubular insert introduced to mimic the presence of a bone. Test results indicated that, for the range of gels considered in this study, softer gels, with Shore OOO hardness in the range of 32.6 ± 0.9 to 34.4 ± 2.0, displayed an impact behavior comparable to experiments with actual hand specimens, thus, making them the most suitable gel grades to represent soft tissues in surrogate hands.

Finally, the current study evaluated the impact behavior of synthetic gels subject to one level of impact energy (8–10 Joules) with a relatively small-diameter impactor. Additional evaluations would be necessary to determine the dynamic behavior of candidate gels subjected to higher energies in the range of 15 to 20 Joules and for larger impacted areas. Additional laboratory work is currently underway to determine the hyperelastic parameters of candidate gels. These parameters are needed to define constitutive models that can be used for computational simulation models of hands subjected to low-velocity impacts. The ultimate objective is to obtain a calibrated and validated model with reliable predicting capabilities that could minimize the need for experimental work.

## Figures and Tables

**Table 1:** Gel identification, densities, and suggested applications. Note: # corresponds to No.

Gel #	Density [Kg/m^3^]	Suggested for Simulating [34]:
0	880.38	Tissues like thigh muscles, biceps, and back muscles.
1	936.48	Neck muscles, healthy skin, liver, heart.
2	923.47	Fattier tissue found around 2 cm below the dermis, skin, and muscles, lung tissue.
3	981.63	Uterus tissue, extremely fatty tissue.
4	834.34	Breast tissue, intestinal tissue, and subcutaneous fat.
5	898.45	Blood clots and brain tissue.

**Table 2:** Summary of hardness test results.

Gel #	Type OOO Hardness [35]		
	Avg. (SD)	COV	Ratio
0	51.5 (1.3)	2.5%	1.00
1	43.2 (1.4)	3.2%	0.84
2	40.2 (2.2)	5.5%	0.78
3	34.4 (2.0)	5.8%	0.67
4	32.6 (0.9)	2.8%	0.63
5	24.9 (1.6)	6.4%	0.48

Note: # corresponds to No.; SD: Standard Deviation; COV: Coefficient of Variation.

**Table 3:** Summary of firmness test results.

Gel #	Ø 12.7 mm Probe			Ø 3.0 mm Probe		
	[N] *	[N] ^1^	Diff. [%] *^,1^	Ratio ^1^	[N] ^2^	Ratio ^1,2^
0	6.73	8.95 (0.19)	33.0%	1.00	1.40 (0.08)	6.4
1	4.20	4.23 (0.17)	0.8%	0.47	0.85 (0.01)	5.0
2	3.02	3.15 (0.10)	4.3%	0.35	0.65 (0.01)	4.8
3	2.23	2.41 (0.13)	8.2%	0.27	0.43 (0.01)	5.6
4	1.76	1.95 (0.10)	11.1%	0.22	0.42 (0.01)	4.7
5	1.25	1.29 (0.12)	3.6%	0.14	0.26 (0.01)	5.0

Note: # corresponds to No; * Reported in grams force (gf) by the manufacturer, converted to Newtons [34]; ^1^ measured in this study; ^2^ measured in this study. SD: Standard Deviation.

## Data Availability

The data presented in this study are available upon reasonable request to the corresponding author.
